# Omicron variant dominance and anti-SARS-CoV-2 vaccination are key determinants for a milder course of COVID-19 in patients with systemic autoimmune rheumatic diseases

**DOI:** 10.1007/s10067-023-06769-4

**Published:** 2023-09-21

**Authors:** Charalampos Papagoras, Nikoleta Zioga, Vasileios Papadopoulos, Nafsika Gerolymatou, Eleni Kalavri, Christos Bounos, Theodora Simopoulou, George E. Fragoulis, Stylianos Panopoulos, Kalliopi Fragiadaki, Gerasimos Evangelatos, Vasiliki-Kalliopi Bournia, Aikaterini Arida, Anastasios Karamanakos, Maria Pappa, Evrydiki Kravvariti, Kleopatra Deftereou, Nikolaos Kougkas, Evangelia Zampeli, Evangelia Kataxaki, Konstantinos Melissaropoulos, Georgia Barouta, Alexandros Panagiotopoulos, Christos Koutsianas, Stamatis-Nick Liossis, Panagiotis Georgiou, Theodoros Dimitroulas, Maria G. Tektonidou, Dimitrios P. Bogdanos, Antonia Elezoglou, Paraskevi V. Voulgari, Petros P. Sfikakis, Dimitrios Vassilopoulos

**Affiliations:** 1 First Department of Internal Medicine, University Hospital of Alexandroupolis, Democritus University of Thrace, Alexandroupolis, Greece; 2 AKESIOS Dialysis Center, Xanthi, Greece; 3 Department of Rheumatology, School of Health Sciences, Faculty of Medicine, University of Ioannina, Ioannina, Greece; 4 Department of Rheumatology, Asklepieion General Hospital, Voula, Athens, Greece; 5 Clinic of Rheumatology and Clinical Immunology, University Hospital of Larissa, Larissa, Greece; 6 1st Department of Propedeutic Internal Medicine, Joint Academic Rheumatology Program, School of Medicine, National and Kapodistrian University of Athens, Athens, Greece; 7 Fourth Department of Internal Medicine, Aristotle University of Thessaloniki, Thessaloniki, Greece; 8 Rheumatology Department, Iaso Hospital, Athens, Greece; 9 Rheumatology Department, General Hospital Elefsinas Thriaseio, Athens, Greece; 10 Department of Rheumatology, Agios Andreas Hospital, Patras, Greece; 11 Private Rheumatology Office, Karditsa, Greece; 12 Clinical Immunology-Rheumatology Unit, 2nd Department of Medicine and Laboratory, Joint Academic Rheumatology Program, School of Medicine, National and Kapodistrian University of Athens, Hippokration General Hospital, 114 Vass. Sophias Ave, 115 27 Athens, Greece; 13 Division of Rheumatology, Department of Internal Medicine, University of Patras Medical School, Patras, Greece

**Keywords:** Anti-SARS-CoV-2 vaccine, Autoimmune rheumatic disease, COVID-19, Mortality, Omicron variant

## Abstract

**Introduction:**

This study aimed to determine whether the introduction of anti-SARS-CoV-2 vaccines and the dominance of the omicron variant had a significant impact on the outcome of COVID-19 in patients with systemic autoimmune rheumatic diseases (SAIRDs).

**Methods:**

Using data entered to the Greek Rheumatology Society COVID-19 registry, we investigated the incidence of hospitalization and death due to COVID-19, during the successive periods of the pandemic according to the prevalent strain (wild-type, Alpha, Delta, Omicron) in vaccinated and unvaccinated patients. Variables independently associated with hospitalization and death were explored using multivariate regression analyses, while Kaplan–Meier curves were used to depict survival data.

**Results:**

From August 2020 until June 30, 2022, 456 cases (70.2% females) of COVID-19 with a mean age (± SD) of 51.4 ± 14.0 years were reported. In unvaccinated patients, the proportions of hospitalization and death were 24.5% and 4%, compared to 12.5% and 0.8% in the vaccinated group (*p* < 0.001 for both comparisons). The rates of hospitalization for the wild-type, Alpha, Delta, and Omicron periods were 24.7%, 31.3%, 25.9%, and 8.1% respectively (*p* < 0.0001), while the case fatality rates were 2.7%, 4%, 7%, and 0%, respectively (*p* = 0.001). Using multivariable regression analysis, factors independently associated with hospitalization were infection by a non-Omicron variant, being non-vaccinated, exposure to rituximab, older age, and respiratory and cardiovascular disease. Independent predictors for death were contracting COVID-19 during the Alpha or Delta period, pulmonary disease, and older age, while being vaccinated was protective.

**Conclusions:**

In this 2-year analysis, the rates of hospitalization and death among patients with SAIRDs have declined significantly. Vaccination and the dominance of the Omicron variant appear to be the major determinants for this shift.

**Table:** 

**Key points** *• During the late phase of the pandemic, the proportion of severe COVID-19 cases, defined as requiring hospitalization or resulting in death, in patients with systemic autoimmune rheumatic diseases has declined.* *• Anti-SARS-CoV-2 vaccination and the dominance of the Omicron strain are the key factors that have independently contributed to this shift.*

**Supplementary Information:**

The online version contains supplementary material available at 10.1007/s10067-023-06769-4.

## Introduction

Coronavirus disease 2019 (COVID-19) is a global pandemic that has challenged the healthcare systems worldwide. It also caused a significant concern over the course of the disease in immunocompromised patients, such as those with systemic autoimmune inflammatory rheumatic diseases (SAIRDs) [1, 2]. This led to the establishment of national and international registries, which provided early insights about the disease characteristics and predictors for adverse outcomes in this patient population [3–8].

Although by end 2022, COVID-19 still remained prevalent, the severity of the disease in the general population had considerably decreased compared to earlier phases of the pandemic. This is due to infection-acquired, vaccine-induced and hybrid immunity against SARS-CoV-2, as well as the latest dominance of the Omicron virus strain, which is thought to cause milder disease [9, 10]. Preliminary evidence suggests that COVID-19 runs a milder course during the later phases of the pandemic among patients with SAIRDs, as well [11]. However, it is not clear whether this is due to previously acquired anti-SARS-CoV-2 immunity, to infection by the Omicron strain or both.

We analyzed data captured in the Greek Rheumatology Society COVID-19 registry since its establishment until well into the Omicron-dominated phase of the pandemic to investigate whether there has been a shift in the severity of COVID-19 and to identify the factors that had a significant impact on disease severity in patients with SAIRDs, focusing on the effects of vaccination and dominant strain.

## Materials and methods

Since March 2020 the Greek Rheumatology Society invited Greek rheumatologists to report cases of COVID-19 in patients with SAIRDs through a web-based anonymized case- report form. Patients were included in the registry if they had an existing diagnosis of a systemic inflammatory rheumatic disease according to the reporting physician and had contracted SARS-CoV-2 infection confirmed with a polymerase chain reaction test or a rapid antigen test. Patients were excluded if they had only non-inflammatory musculoskeletal diseases, such as osteoarthritis, or the outcome of COVID-19 was still pending or unknown. The collected data included patient age, sex, characteristics of COVID-19 (including date of diagnosis, symptoms, hospitalization, recovery or death, use of antiviral treatments), details on the anti-SARS-CoV-2 vaccination (dates and type of vaccine), SAIRD characteristics including immunomodulatory treatments, co-morbidities, and smoking habits. For the analysis, less commonly reported SAIRDs were grouped together: Sjögren’s syndrome, idiopathic inflammatory myopathies, and mixed or undifferentiated connective tissue (CTD) disease were grouped as “other CTD,” while miscellaneous inflammatory conditions, including polymyalgia rheumatica (without giant cell arteritis), autoinflammatory syndromes, and sarcoidosis, were grouped as other inflammatory diseases, “other ID”.

Since the Greek state issued early in the pandemic recommendations for the management of patients with COVID-19, including criteria for hospital admission, we used hospitalization and death as markers of severe disease. Moreover, we divided patients into vaccinated, i.e., those who had received at least one dose of anti-SARS-CoV-2 vaccine before contracting COVID-19, and unvaccinated, i.e., those who had received no vaccine dose. Finally, we divided the course of the pandemic into 4 periods according to the dominance of each virus strain in Greece as follows: wild-type-dominant period until week 02, 2021; Alpha (B.1.1.7) variant-dominant period from week 03, 2021 until week 26, 2021; Delta (B.1.617.2) variant-dominant period from week 27, 2021 until week 50, 2021; and Omicron (BA.1 up to BA.4/BA.5) variant-dominant period from week 51, 2021 until 30 June 2022 that is the limit for the current analysis [12, 13]. Cases of re-infection in unvaccinated patients were excluded to remove the effect of natural immunization on the outcome of re-infection.

Descriptive statistics are provided as means with the relevant standard errors, or percentages, for scale and discrete parameters, respectively. Survival data are presented as medians and accompanied by their 95% confidence intervals. Comparisons of two sets of discrete and continuous data were performed using chi-square test (or, alternatively, Fisher’s exact test if expected values were < 5 in > 25% of cells) and Student’s t-test, respectively (if *N* > 30). To test for mean differences among at least three groups, one-way ANOVA was preferred. To explore the potential independent correlations between survival data (occurrence of infection, hospitalization, death) and independent variables, univariate and multivariate analyses were performed using Cox proportional hazards regression analysis. Kaplan–Meier (KM) curves were used to depict survival data; the log-rank test was used to determine the univariate significance of the study variables. Similarly, to explore differences in hospitalization and case fatality rate, binary logistic regression was used for univariate and multivariate analysis.

All reported *p* values are two sided. The level of statistical significance was set to *p* = 0.05. Statistical analysis and visualization of KM curves was performed with the use of IBM SPSS Statistics software, version 26.0, for Windows. The Review Manager version 5.3 (RevMan, Copenhagen: The Nordic Cochrane Centre, The Cochrane Collaboration; 2014) was used to illustrate forest plots.

Using the UK Health Research Authority decision tool, the registry is not classified as a human subjects’ research study. Additionally, due to the anonymized and non- interventional nature of the survey, ethics approval and patient consent were not required.

## Results

### Patient characteristics

Since the first case reported in August 2020 up to June 30th, 2022, 456 cases of COVID-19 in patients with SAIRDs fulfilling the above-mentioned inclusion and exclusion criteria were recorded in the registry. They were 320 females (70.2%) and 136 males (29.8%) with a mean age (± SD) of 51.4 ± 14.0 years (Table 1). The most common underlying rheumatic diseases were rheumatoid arthritis (*n* = 96, 21.1%), systemic lupus erythematosus (*n* = 77, 16.9%), psoriatic arthritis (*n* = 71, 15.6%), axial spondyloarthritis (*n* = 63, 13.8%), systemic sclerosis (*n* = 44, 9.6%), and vasculitis (*n* = 42, 9.2%). During each dominance period, i.e., wild-type, Alpha, Delta, and Omicron, 73, 100, 86, and 197 cases were reported, respectively.

**Table 1:** Patient characteristics according to dominant strain (*n* = 456)

	Whole population (*n* = 456)		Wild-type (*n* = 73)	Alpha (*n* = 100)	Delta (*n* = 86)	Omicron (*n* = 197)	*P*^a^
SARS-CoV-2 strain data							
Time interval of dominance			w33, 2020 to w02, 2021	w03, 2021 to w26, 2021	w27, 2021 to w50, 2021	w51, 2021 to w26, 2022	
Duration of dominance (days)			160	168	168	195	
Patient data							
Patients infected, *n*		456	73	100	86	197	
Patients hospitalized, *n*, %		87/456 19.1%	18/73 24.7%	31/100 31%	22/86 25.6%	16/197 8.1%	< 0.0001^b^
In-hospital deaths, *n* %		10/456 2.19%	2/73 2.7%	3/100 3%	5/86 5.8%	0/197 0%	0.198^c^
Total deaths, *n* %		12/456 2.63%	2/73 2.7%	4/100 4%	6/86 7%	0/197 0%	0.001^c^
Vaccination status at time of infection, *n* (%)							
Not vaccinated		151 (33.1%)	71 (97.3%)	30 (57.7%)	18 (22.2%)	32 (16.4%)	< 0.001^c^
Vaccinated (1 dose)		23 (5%)	1 (1.4%)	14 (26.9%)	4 (5.0%)	4 (2.1%)	
Vaccinated (2 doses)		108 (23.7%)	0 (0.0%)	6 (11.5%)	52 (65.0%)	50 (25.6%)	
Vaccinated (3 doses)		112 (24.6%)	0 (0.0%)	2 (3.8%)	6 (7.5%)	104 (53.3%)	
Vaccinated (4 doses)		5 (1.1%)	0 (0.0%)	0 (0.0%)	0 (0.0%)	5 (2.6%)	
Missing data		57 (12.5%)	1	48	6	2	
Sex							
Female		320 (70.2%)	52 (71.2%)	73 (73%)	62 (72.1%)	133 (67.5%)	0.704^b^
Male		136 (29.8%)	21 (28.8%)	27 (27%)	24 (27.9%)	64 (32.5%)	
Age							
Mean ± SE		51.4 ± 14	51.6 ± 1.5	52.6 ± 1.4	50.5 ± 1.6	51.5 ± 1.0	0.626^d^
SAIRD							
RA		96 (21.1%)	11 (15.1%)	27 (27%)	19 (22.1%)	39 (19.8%)	0.251^b^
SpA		142 (31.1%)	28 (38.4%)	30 (30%)	22 (25.6%)	62 (31.5%)	0.407^b^
Other arthritis		14 (3.1%)	2 (2.7%)	3 (3%)	3 (3.5%)	6 (3%)	1.000^c^
SLE/APS		83 (18.2%)	14 (19.2%)	9 (9%)	20 (23.3%)	40 (20.3%)	0.126^c^
SSc		44 (9.6%)	7 (9.6%)	13 (13%)	7 (8.1%)	17 (8.6%)	0.621^b^
Other CTD		32 (7%)	2 (2.7%)	8 (8%)	5 (5.8%)	17 (8.6%)	0.363^b^
Vasculitis		42 (9.2%)	11 (15.1%)	7 (7%)	8 (9.3%)	16 (8.1%)	0.287^b^
Other ID		9 (2%)	0 (0.0%)	4 (4%)	2 (2.3%)	3 (1.5%)	0.294^c^
Overlapping		6 (1.3%)	2 (2.7%)	1 (1%)	0 (0%)	3 (1.5%)	0.840^c^
SAIRD treatment, *n*/*N* (%)							
No treatment			7/73 (9.6%)	14/99 (14.1%)	5/85 (5.9%)	3/196 (1.5%)	< 0.001^b^
Glucocorticoids			44/73 (60.1%)	64/99 (64.6%)	44/85 (51.8%)	80/196 (40.8%)	0.004^b^
csDMARDs^e^			40/73 (54.8%)	59/99 (59.6%)	41/85 (48.2%)	112/196 (57.1%)	0.440^b^
Immunosuppressants^f^			7/73 (9.6%)	7/99 (7.1%)	10/85 (11.8%)	35/196 (17.9%)	0.045^b^
Biologics							
TNFi			15/73 (20.5%)	27/99 (27.3%)	27/85 (31.8%)	61/196 (31.1%)	0.333^b^
IL-6i			5/73 (6.8%)	6/99 (6.1%)	1/85 (1.2%)	6/196 (3.1%)	0.161^c^
IL-17/23/12i			8/73 (11.0%)	2/99 (2.0%)	2/85 (2.4%)	10/196 (5.1%)	0.049^c^
Anti-CD20			2/73 (2.7%)	3/99 (3.0%)	10/85 (11.8%)	21/196 (10.7%)	0.011^c^
Belimumab			0/73 (0.0%)	2/99 (2.0%)	6/85 (7.1%)	6/196 (3.1%)	0.076^c^
JAKi			1/73 (1.4%)	3/99 (3.0%)	3/85 (3.5%)	6/196 (3.1%)	0.916^c^
Other			6/73 (8.2%)	3/99 (3.0%)	3/85 (3.5%)	8/196 (4.1%)	0.415^c^
Comorbidities, *n*/*N* (%)							
Diabetes mellitus			5/73 (6.8%)	8/99 (8.1%)	1/83 (1.2%)	24/196 (12.2%)	0.022^b^
Arterial hypertension			20/73 (27.4%)	27/99 (27.3%)	13/83 (15.7%)	65/196 (33.2%)	0.030^b^
Malignancy			4/73 (5.5%)	1/99 (1.0%)	3/83 (3.6%)	1/196 (0.5%)	0.025^c^
Pulmonary diseases			11/73 (15.1%)	17/99 (17.2%)	15/83 (18.1%)	38/196 (19.4%)	0.966^c^
Cardiovascular diseases			3/73 (4.1%)	8/99 (8.1%)	8/83 (9.6%)	28/196 (14.3%)	0.071^b^
Obesity			15/73 (20.5%)	24/99 (24.2%)	6/83 (7.1%)	19/196 (9.7%)	< 0.001^b^
Smoking ever			33/66 (50.0%)	47/97 (48.5%)	47/74 (63.5%)	113/169 (66.9%)	0.009^b^
Other/unknown			0/73 (0.0%)	5/99 (5.1%)	2/83 (2.4%)	3/196 (1.5%)	0.129^c^
None			27/73 (37.0%)	33/99 (33.3%)	29/83 (34.9%)	70/196 (35.7%)	0.963^b^

^a^*P* value for the comparison of means (for continuous variables) or proportions (for discrete variables) among the four time periods of dominance of the different virus strains

^b^Chi-square test

^c^Fisher’s exact test

^d^One-way ANOVA

^e^Includes hydroxychloroquine, methotrexate, leflunomide, azathioprine, sulfasalazine, and apremilast

^f^Includes cyclophosphamide, mycophenolate, and cyclosporine

*RA*, rheumatoid arthritis, *SpA*, spondyloarthritis; *SLE*, systemic lupus erythematosus; *APS*, antiphospholipid syndrome; *SSc*, systemic sclerosis; *CTD*, connective tissue disease; *ID*, inflammatory disease; *csDMARDs*, conventional synthetic disease-modifying anti-rheumatic drugs; *TNFi*, tumor necrosis factor inhibitor; *IL*, interleukin; *JAKi*, Janus kinase inhibitor

At the time of the infection 248 patients had received at least 1 dose of the vaccine (54%), 151 patients had received no vaccine dose (33%), and for 57 patients (13%) their vaccination status was unknown. No patient had received a single-dose vaccine. Throughout the pandemic 87 (19.1%) patients were hospitalized and 12 (2.6%) patients died.

The characteristics of patients who were infected during each dominance period are shown in Table 1. Their demographics as well as the proportions of the underlying SAIRD were comparable across the 4 periods. There were slight differences in the exposure of patients to anti-rheumatic medications with more patients receiving immunosuppressants in the Omicron period, B cell-depleting treatments (during the previous 12 months) in the Delta and Omicron period and fewer patients receiving glucocorticoids during the Omicron period. However, only 1.5% of patients received no immunomodulatory/immunosuppressive treatment during the Omicron period, compared to 9.7%, 14.1%, and 5.9% during the 3 preceding periods, respectively (*p* < 0.001). Regarding co-morbidities, there were also slight differences in the prevalence of diabetes, arterial hypertension, and malignancy across periods, with the greatest difference regarding obesity, which was more frequent among the patients in the wild-type and Alpha period.

### Hospitalization and mortality rates according to the vaccination status

**Fig. 1:**
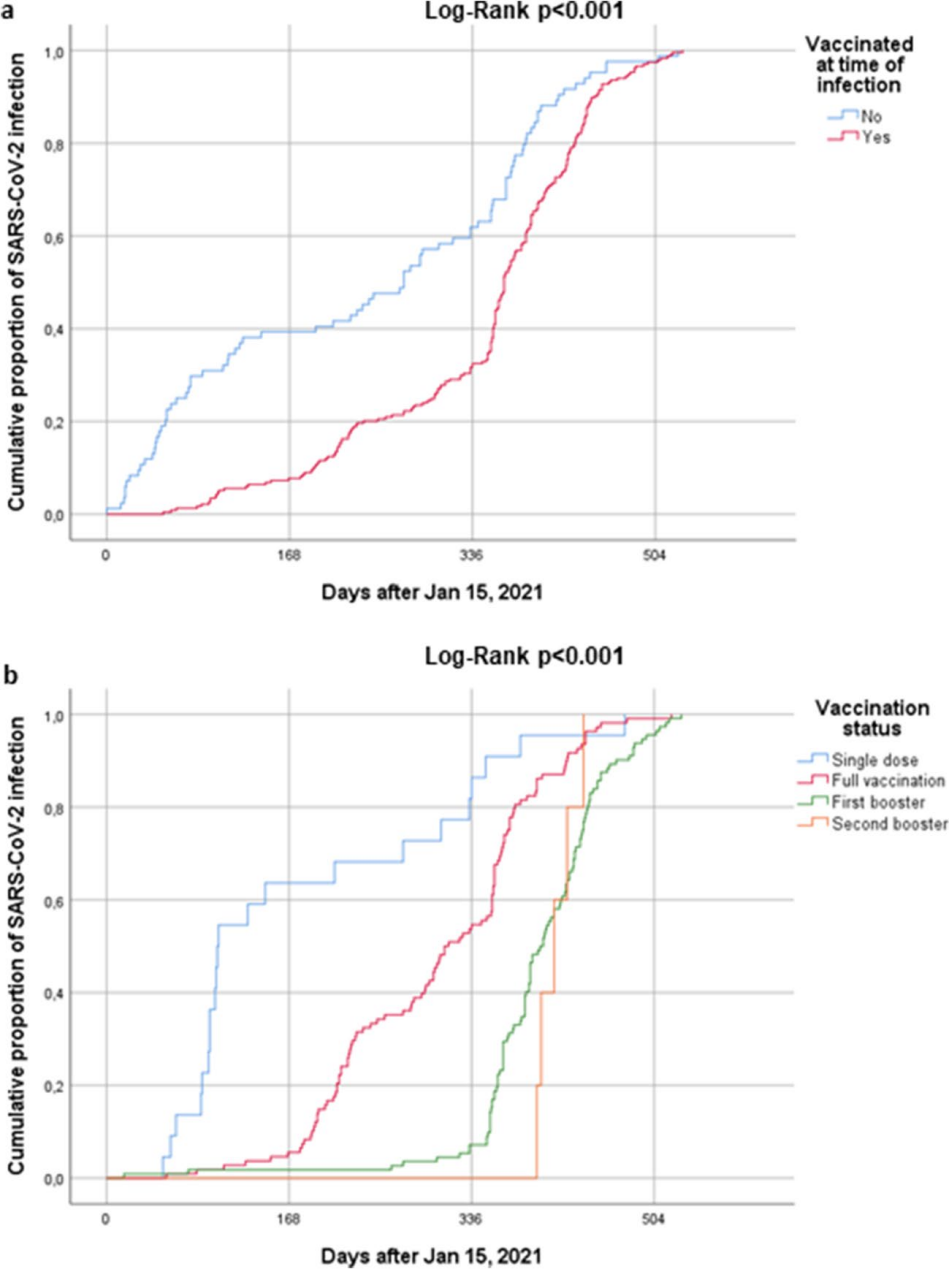
Cumulative proportion of infection according to **a** *Vaccination status (vaccinated vs. non vaccinated).* Patients who were vaccinated at least with a single dose presented an increased median estimate of time to infection (365 days, 95% CI: 356–374 days) compared to unvaccinated patients (273 days, 95% CI: 221–325 days). **b** *Number of vaccine doses received (single dose vs. full vaccination, 1st booster, 2nd booster).* Time to infection was 102 days (95% CI: 68–136 days) in patients who received a single dose, 311 days (95% CI: 274–348 days) in fully vaccinated patients, 399 days (95% CI: 387–411 days in patients who received one booster dose, and 412 days (95% CI: 386–438 days) in patients who received two booster doses

**Fig. 2:**
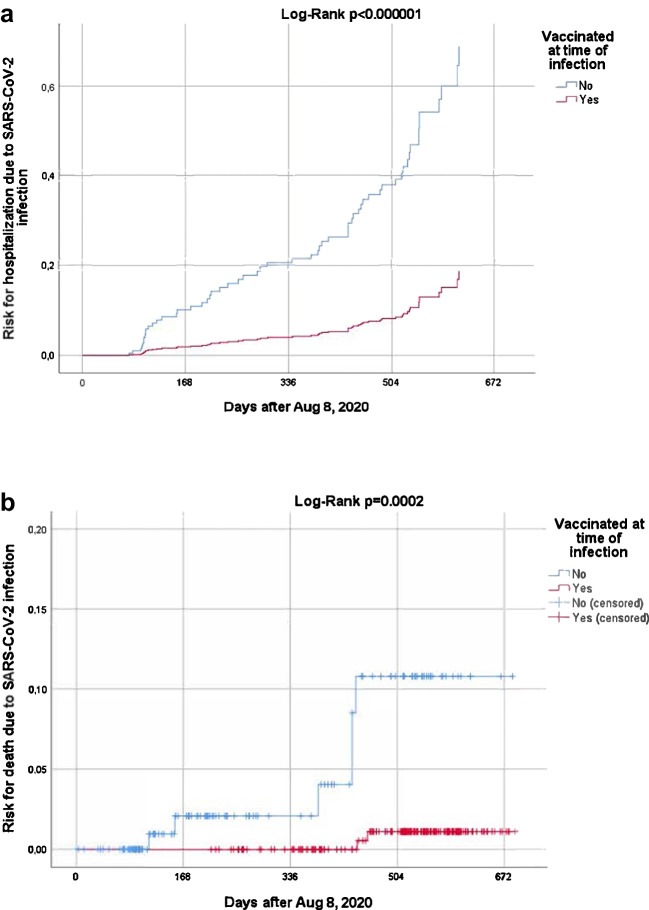
Cumulative risk for hospitalization (**a**) and death (**b**) according to vaccination status, among patients with SARS-CoV-2 infection. For hospitalization, a Cox regression model was used to adjust for all potential confounders as deduced from Table 1 (no treatment received; treatment with glucocorticoids; treatment with immunosuppressants; treatment with IL-17/23/12i; treatment with anti-B-cell regimens; diabetes mellitus; arterial hypertension; malignancy; obesity; smoking)

As expected, the proportion of vaccinated patients gradually increased from the wild- type period, when 1 patient was vaccinated, up to the Omicron period, when only 16.4% of patients had received no vaccine dose (Table 1). As shown in Fig. 1A and 1B, following the introduction of the anti-SARS-CoV-2 vaccine in Greece in January 2021, the cumulative proportion of infection was significantly higher among vaccinated patients particularly after exposure to multiple vaccine doses (*p* < 0.001 for all comparisons). The cumulative hospitalization rate in vaccinated patients was 12.5% compared to 24.5% in unvaccinated patients. After adjustment for all potential confounders as deduced from Table 1, the cumulative risk for hospitalization due to COVID-19 was significantly lower in vaccinated patients compared to unvaccinated ones (*p* < 0.001, Fig. 2A). Moreover, the cumulative mortality rate was 0.8% in vaccinated patients, which was also significantly lower compared to 4% in unvaccinated (*p* < 0.001, Fig. 2B).

### Hospitalization and mortality rates according to the dominant viral strain period

**Fig. 3:**
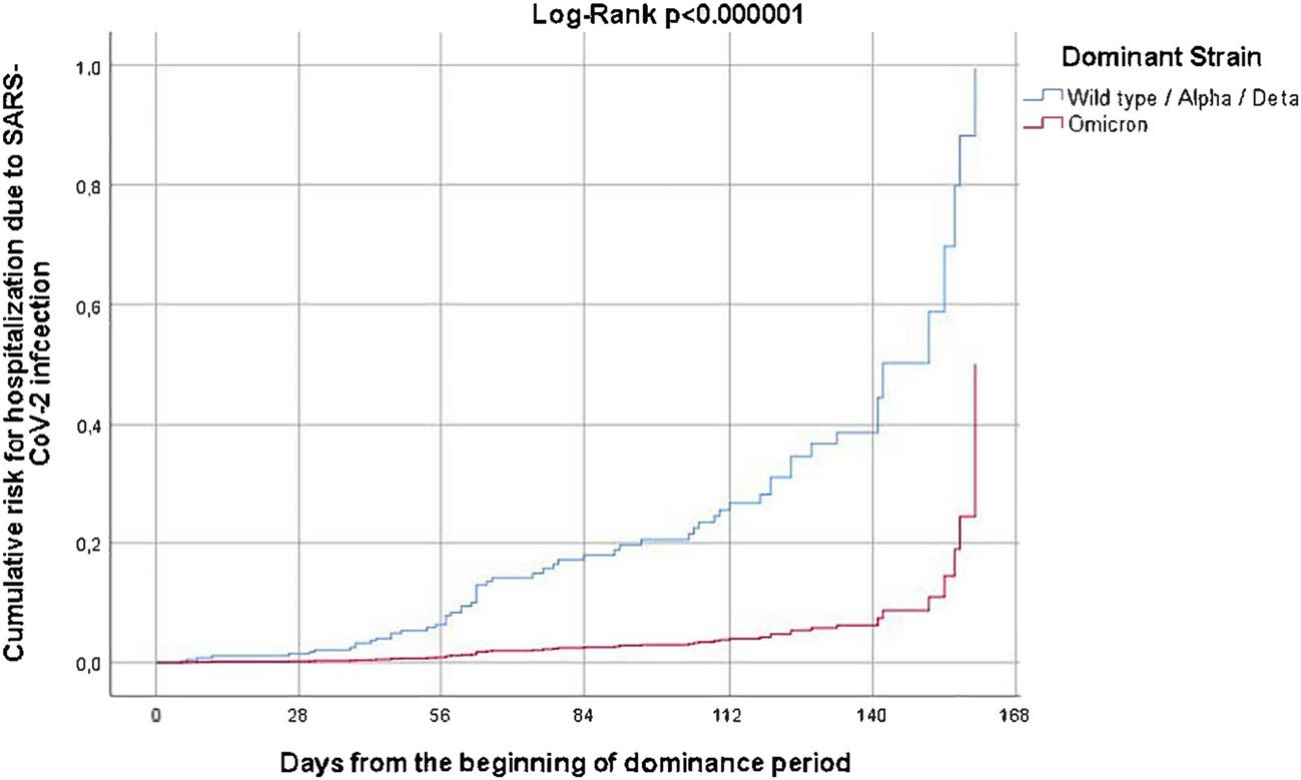
Cumulative risk for hospitalization according to the dominant SARS-CoV-2 strain (Omicron vs. others) derived from Cox regression models after adjustment for all potential confounders (vaccination, no treatment received; treatment with glucocorticoids; treatment with immunosuppressants; treatment with IL-17/23/12i; treatment with anti-B-cell regimens; diabetes mellitus; arterial hypertension; malignancy; obesity; smoking; Table 1)

Although 197 (43.2%) infections occurred during the Omicron period, only 8.1% of patients were hospitalized compared to 24.7%, 31.3%, and 25.9% of patients during the wild-type, Alpha, and Delta period, respectively (*p* < 0.001, after adjustment for all potential confounders shown in Table 1  (Fig. 3). Moreover, no deaths occurred during the Omicron period compared to 2 (2.7%), 4 (4%), and 6 (7%) deaths during the 3 preceding periods, respectively, resulting in a significantly lower case fatality rate during the Omicron period (*p* = 0.001, Table 1).

Since oral antiviral treatments, namely, molnupiravir and nirmatrelvir/ritonavir, which were available only during the Omicron period, may have contributed to the above differences, we compared the proportion of hospitalization according to the exposure to those drugs during the Omicron period (*n* = 197). Among 37 patients who received antiviral therapies, the hospitalization rate was 8.1% (3/37) which was not different from the rate in patients who did not take any antiviral therapy (*p* > 0.05).

### Factors associated with serious outcomes

**Fig. 4:**
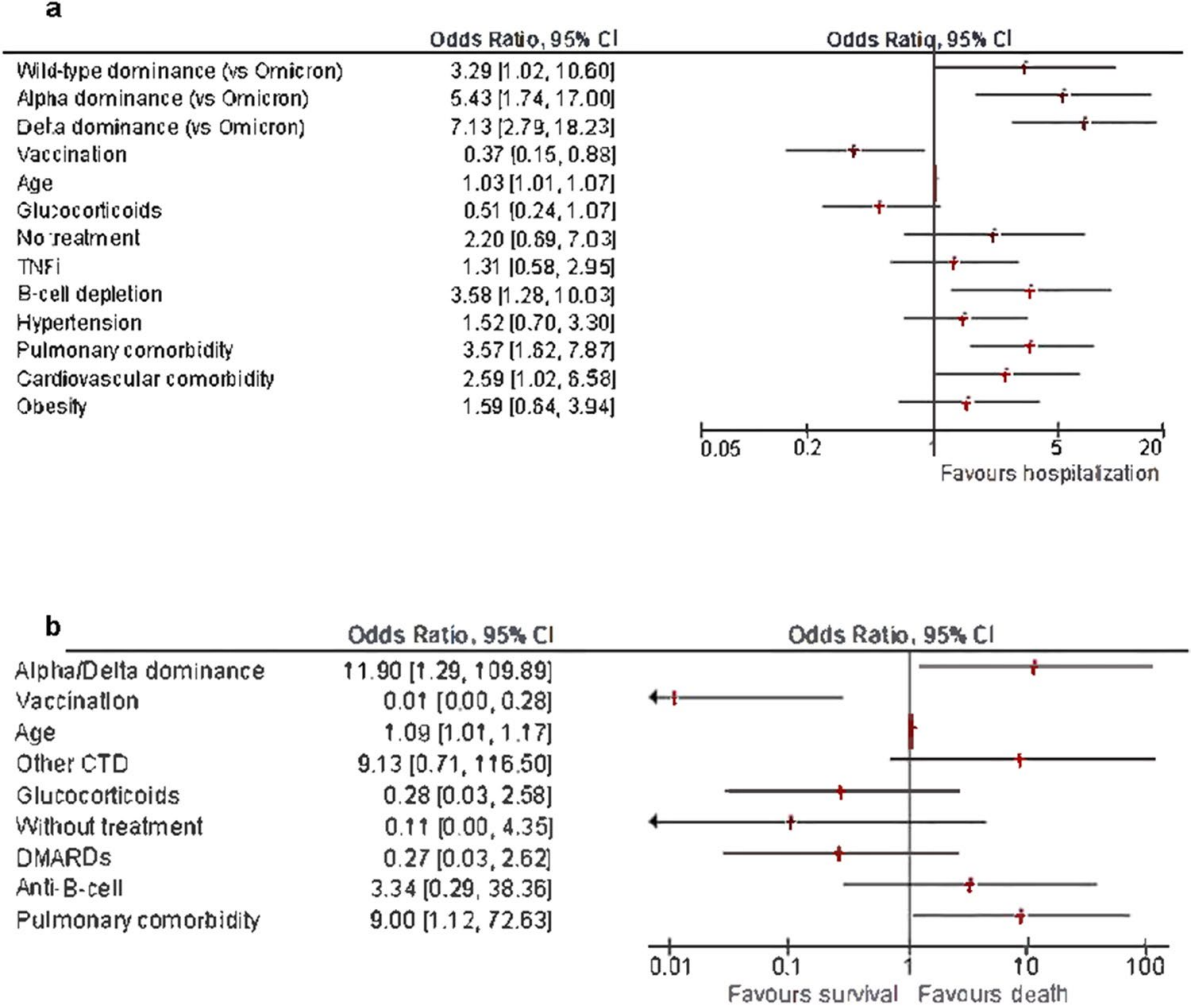
**a** Forest plot depicting odds ratios of all included independent variables as derived from multivariate binary regression analysis data of Supplementary Table 1; baseline odds ratio for strain (OR = 1) corresponds to Omicron. **b** Forest plot depicting odds ratios of all included independent variables as derived from multivariate binary regression analysis data of Supplementary Table 2

By univariate analysis, the factors associated with hospitalization throughout the pandemic are shown in Supplementary Table 1. By multivariable regression analysis, contracting COVID-19 during the wild-type, Alpha, and Delta periods was associated with significantly increased odds ratios (OR) for hospitalization compared to the Omicron period [OR for wild type 3.29, 95% confidence interval (CI) 1.02–10.6; OR for Alpha 5.43, 95% CI 1.74– 17.0; OR for Delta 7.13, 95% CI 2.79–18.23). Being exposed to B cell-depleting therapies was also associated with an increased OR for hospitalization (3.58, 95% CI 1.28–10.04), as well as older age (OR 1.035, 95% CI 1.006–1.065), pulmonary (OR 3.57, 95% CI 1.62–7.87), and cardiovascular disease (OR 2.59, 95% CI 1.02–6.58). Conversely, the risk for hospitalization was significantly decreased with vaccination (OR 0.37, 95% CI 0.15–0.88, Supplementary Table 1 and Fig. 4A).

Factors associated with death throughout the pandemic are shown in Supplementary Table 2. In multivariable regression analysis, independent predictors for mortality were contracting COVID-19 during the Alpha or Delta period [OR 11.9, 95% CI 1.3–109.9), older age (OR 1.09, 95% CI 1.008–1.172), and pulmonary disease (OR 9, 95% CI 1.1–72.6). Conversely, being vaccinated was associated with a significantly lower risk for death (OR 0.011, 95% CI 0.001–0.28, Supplementary Table 2 and Fig. 4B).

## Discussion

We performed an analysis of the data collected in the Greek Rheumatology Society COVID-19 Registry over 2 years to assess and compare the most serious outcomes of infection during the different phases of the pandemic. Our results expand and replicate our previous preliminary analysis of the registry regarding the benefits of anti-SARS-CoV-2 vaccination in patients with SAIRDs [14]. Indeed, in line with other studies [15, 16], vaccination seems to prevent clinical infection as shown by the cumulative proportion of COVID-19 in vaccinated versus unvaccinated patients. Moreover, vaccination significantly decreased the rates not only of hospitalization but also mortality regardless of the prevailing SARS-CoV-2 strain, underlying rheumatic disease and its treatment, as well as co-morbidities. According to the multivariable regression analysis, having received at least one dose of the vaccine decreased the risk for hospitalization by 63% and the risk for death by 99%. Compared to population studies, the latter figure may appear somewhat inflated despite statistical adjustment [17, 18]. This is possibly because the highest proportion of vaccinated patients was attained in the Omicron period during which no deaths were reported to the registry.

At the time of data lock for this analysis, the updated vaccines against the BA.4/BA.5 Omicron variants had not been available in Greece, and therefore patients had received the older vaccines that their effectiveness against the Omicron variant had been questioned [19, 20]. Considering that during the Omicron period over 50% of patients had received a booster dose and that the more the vaccine doses the lower the cumulative proportion of COVID-19, our results suggest that booster doses of the older vaccines enhanced protection against the Omicron variant as well. This is in line with reports, both in the general population and immunocompromised patients [20–24].

The second most important independent predictive factor for serious outcomes, namely, hospitalization and death, was the dominant virus strain. Indeed, while the rate of case reporting to the registry doubled during the Omicron period, the hospitalizations and deaths declined. Taking the Omicron period as referent, infection with the Alpha or Delta strain had a 5.4-fold or sevenfold increased risk for hospitalization, respectively. Regarding the outcome of death, infection during the Alpha or Delta periods was associated with a 12-fold greater risk compared to the preceding (i.e., the wild-type) or the succeeding (i.e., the Omicron) periods. This agrees with observations in the general population, as well as observations in patients with SAIRD early in the Omicron period [10, 11, 24].

The rest of the factors determining serious outcomes included an underlying respiratory or cardiovascular disease and exposure to rituximab within the previous 12 months, while no other immunomodulatory treatment was independently associated with hospitalization or death. Respiratory and cardiovascular diseases are recognized predictors of hospitalization and death in patients with SAIRDs [3, 4]. Moreover, rituximab has both been associated with more severe course of COVID-19 [24–27], as well as with blunted humoral, but not cellular responses to the vaccines [28, 29]. Of note, while in this study there was a 3.6 times higher risk for hospitalization with rituximab use, accounting for 11.5 excess hospitalizations in every 100 infected patients on rituximab, the risk for death was not significantly increased. Finally, although elderly patients have worse COVID-19 outcomes, in multivariate analysis the effect of age was the least important compared to the other factors, such as vaccination status, in line with observation in large populations [30].

A confounding factor that could not be adjusted for the whole length of the pandemic is the use of antiviral drugs by outpatients, i.e., molnupiravir and nirmatrelvir/ritonavir, because they were available only during the Omicron period. In clinical trials these drugs have shown efficacy in preventing hospitalization and death of adults affected by COVID-19 at high risk for progression [31, 32]. Moreover, in a series of patients with SAIRDs, no patient treated with oral antivirals was hospitalized due to COVID-19 [33]. However, in the registry population only 19% of the Omicron period patients received those drugs with no difference in hospitalization rates between those who had received them versus those who had not. Therefore, in our sample the more favorable disease course during the Omicron period cannot be solely attributed to the availability of antiviral treatments. However, due to small patient numbers it was difficult to assess the true effect of those drugs in COVID-19 outcomes during the Omicron period.

Another limitation of the study is the voluntary character of the registry, so that it could not capture most Greek patients with SAIRDs who contracted COVID-19. However, the identification of adverse risk factors common with other studies, such as rituximab use and respiratory and cardiovascular diseases, suggests that the cases reported in the registry were representative of the actual patient population and similar to other registries. Moreover, plenty of patients, particularly hospitalized ones, were not treated for COVID-19 by the reporting rheumatologist. Therefore, there is a lack of information about in-hospital treatments and how they evolved over time, which may have also influenced the outcomes of hospitalized patients.

## Conclusions

Since the outset of the pandemic, the clinical features of COVID-19 in patients with SAIRDs have changed, with fewer hospitalizations and deaths during the latest phase, similar to the general population. This overview of the Greek COVID-19 registry shows that the major determinants for this shift was the acquisition of large-scale anti-SARS-CoV-2 immunity, particularly through vaccination, as well as the dominance of the Omicron virus strain, which appears to cause a less severe disease.

### Supplementary Information

Below is the link to the electronic supplementary material.

Supplementary file1 (PDF 539 KB)

## Data Availability

The datasets generated and/or analyzed during the current study are not publicly available as they represent patient-level data, but are available from the corresponding author on reasonable request.
