# Supplementary material for: Omicron variant dominance and anti-SARS-CoV-2 vaccination are key determinants for a milder course of COVID-19 in patients with systemic autoimmune rheumatic diseases
Source: Clin Rheumatol. 2023 Sep 21;42(12):3375–85. doi: 10.1007/s10067-023-06769-4 (PMC10640401; doi:10.1007/s10067-023-06769-4)
Supplement: Supplementary file 1 — Supplementary file1 (PDF 539 KB) [file 10067_2023_6769_MOESM1_ESM.pdf]

Supplementary Material

Omicron variant dominance and anti-SARS-CoV-2 vaccination are key determinants for a milder course of COVID-19 in patients with systemic autoimmune autoimmune rheumatic diseases

For Clinical Rheumatology

Charalampos Papagoras[1], Nikoleta Zioga[1], Vasileios Papadopoulos[2], Nafsika Gerolymatou[3], Eleni Kalavri[4], Christos Bounos[4], Theodora Simopoulou[5], George E. Fragoulis[6] , Stylianos Panopoulos[6], Kalliopi Fragiadaki[6], Gerasimos Evangelatos[6], Vasiliki-Kalliopi Bournia[6], Aikaterini Arida[6], Anastasios Karamanakos[6], Maria Pappa[6], Evrydiki Kravvariti[6], Kleopatra Deftereou[7], Nikolaos Kougkas[7] , Evangelia Zampeli[8], Evangelia Kataxaki[9], Konstantinos Melissaropoulos[10] , Georgia Barouta[11] , Alexandros Panagiotopoulos[12], Christos Koutsianas[12], Stamatis-Nick Liossis[13], Panagiotis Georgiou[10] , Theodoros Dimitroulas[7], Maria G Tektonidou[6], Dimitrios P Bogdanos[5] , Antonia Elezoglou[4] , Paraskevi Voulgari[3] , Petros P Sfikakis[6], Dimitrios Vassilopoulos[12]

1. First Department of Internal Medicine, University Hospital of Alexandroupolis, Democritus University of Thrace, Alexandroupolis, Greece

2. AKESIOS Dialysis Center, Xanthi, Greece

3. Department of Rheumatology, School of Health Sciences, Faculty of Medicine, University of Ioannina, Ioannina, Greece

4. Department of Rheumatology, Asklepieion General Hospital, Voula, Athens, Greece

5. Clinic of Rheumatology and Clinical Immunology, University Hospital of Larissa, Larissa, Greece

6. Joint Academic Rheumatology Program, 1st Department of Propedeutic Internal Medicine, School of Medicine, National and Kapodistrian University of Athens, Athens, Greece

7. Fourth Department of Internal Medicine, Aristotle University of Thessaloniki, Thessaloniki, Greece

8. Rheumatology Department, Iaso Hospital, Athens, Greece

9. Rheumatology Department, General Hospital Elefsinas Thriaseio, Athens, Greece

10. Department of Rheumatology, Agios Andreas Hospital, Patras, Greece

11. Private Rheumatology Office, Karditsa, Greece

12. Joint Academic Rheumatology Program, Clinical Immunology-Rheumatology Unit, 2nd Department of Medicine and Laboratory, National and Kapodistrian University of Athens, Athens, Greece

13. Division of Rheumatology, Department of Internal Medicine, University of Patras Medical School, Patras, Greece

Corresponding author: Prof. Dimitrios Vassilopoulos, School of Medicine, 2nd Department of Medicine and Laboratory, Clinical Immunology-Rheumatology Unit, National and Kapodistrian University of Athens, Hippokration General Hospital, 114 Vass. Sophias Ave.,115 27 Athens, Greece, dvassilop@med.uoa.gr

Supplementary Table 1. Uni- and multi-variable analysis of factors associated with hospitalization						
	**Non-hospitalized (n=369)**	**Hospitalized (n=87)**	**Univariate analysis**	**Multivariate analysis⁋**		
			**p**	**OR**	**±95% CI**	**p**
**Strain**						
Wild-type dominance	**55/367 (15.0%)**	**18/87 (20.7%)**	**<0.0001**	**3.287**	**1.019-10.60**	**0.046**
Alpha dominance	**68/367 (18.5%)**	**31/87 (35.6%)**		**5.434**	**1.737-17.00**	**0.004**
Delta dominance	**63/367 (17.2%)**	**22/87 (25.3%)**		**7.133**	**2.791-18.23**	**<0.0001**
Omicron dominance	**181/367 (49.3%)**	**16/87 (18.4%)**		**1.00**		
**Vaccination**						
No	**120/326 (36.8%)**	**36/65 (55.4%)**	**0.005†**	**0.367**	**0.152-0.884**	**0.025**
Yes	**206/326 (63.2%)**	**29/65 (44.6%)**				
**Vaccination status**						
Not vaccinated	**114/325 (34.0%)**	**37/68 (54.4%)**	**0.014‡**			
Vaccinated (1 dose)	**20/325 (6.0%)**	**4/68 (5.9%)**				
Vaccinated (2 doses)	**93/325 (27.8%)**	**17/68 (25.0%)**				
Vaccinated (3 doses)	**103/325 (30.7%)**	**10/68 (14.7%)**				
Vaccinated (4 doses)	**5/325 (1.2%)**	**0/68 (0.0%)**				
Gender						
Female	261/369 (70.7%)	59/87 (67.8%)	0.593†			
Male	108/369 (29.3%)	28/87 (32.2%)				
**Age (years)**						
Mean ± SE	**49.8 ± 0.7**	**58.0 ± 1.5**	**<0.0001⁂**	**1.035**	**1.006-1.065**	**0.018**
SAIRD						
RA	84 (22.8%)	14 (16.1%)	0.173†			
SpA	121 (32.8%)	21 (24.1%)	0.117†			
Other arthritis	11 (3.0%)	3 (3.4%)	0.533†			
SLE / APS	65 / 5 (19.0%)	13 / 1 (16.0%)	0.855‡			
SSc	32 (8.7%)	12 (13.8%)	0.146†			
Vasculitis	33 (8.9%)	9 (10.3%)	0.684†			
Treatments						
No treatment	**14/368 (3.8%)**	**15/87 (17.2%)**	**<0.0001†**	2.203	0.690-7.042	0.182
Glucocorticoids	**196/350 (56.0%)**	**37/84 (44.0%)**	**0.049†**	0.511	0.244-1.071	0.075
DMARDs	213/369 (57.7%)	42/87 (48.3%)	0.110†			
Immunosuppressants*	44/369 (11.9%)	15/87 (17.2%)	0.184†			
TNFi	**114/369 (30.9%)**	**17/87 (19.5%)**	**0.035†**	1.312	0.584-2.950	0.510
IL-6i	16/369 (4.3%)	2/87 (2.3%)	0.380†			
IL-17/23/12i	20/369 (5.4%)	2/87 (2.3%)	0.222†			
B cell depletion	21/369 (5.7%)	15/87 (17.2%)	0.029†	**3.584**	**1.280-10.04**	**0.015**
JAKi	13/369 (3.5%)	0/87 (0.0%)	0.076†			
Belimumab	11/369 (3.0%)	3/87 (3.4%)	0.820†			

Other	19/369 (5.1%)	1/87 (1.1%)	0.101†			
Comorbidities						
Diabetes mellitus	27/367 (7.4%)	11/87 (12.6%)	0.109†			
Arterial hypertension	90/367 (24.5%)	35/87 (40.2%)	0.003†	1.517	0.697-3.300	0.294
Malignancy	5/367 (1.4%)	4/87 (4.6%)	0.052†			
Pulmonary diseases	49/369 (13.3%)	32/87 (36.8%)	<0.0001†	**3.571**	**1.629-7.874**	**0.002**
Cardiovascular diseases	28/369 (7.6%)	19/87 (21.8%)	<0.0001†	**2.591**	**1.019-6.579**	**0.045**
Obesity	43/369 (11.7%)	21/87 (24.1%)	0.003†	1.592	0.646-3.937	0.312
Smoking ever	193/326 (59.2%)	48/81 (59.3%)	0.993†			
Other/Unknown	10/367 (2.7%)	2/87 (2.3%)	0.824†			
None	143/367 (39.0%)	17/87 (10.6%)	0.001†			

† Chi-square test
‡ Fisher's Exact test
⁑ Log-Rank test
⁂ Student's t-test
⁋ Binary regression
*Includes cyclophosphamide, mycophenolate, cyclosporine
DMARDs include: hydroxychloroquine, methotrexate, leflunomide, azathioprine, sulfasalazine, apremilast

Supplementary Table 2. Uni-and multi-variable analysis of factors associated with mortality						
	**Survived (n=444)**	**Deceased (n=12)**	**Univariable analysis**	**Multivariable analysis⁋**		
			**p**	**OR**	**± 95% CI**	**p**
**Alpha/Delta dominance**						
No	**268/444 (60.4%)**	**2 (16.7%)**	**0.002†**	**11.90**	**1.294-109.9**	**0.029**
Yes	**176/444 (39.6%)**	**10 (83.3%)**				
**Omicron dominance**						
Yes	**197/444 (44.4%)**	**0 (0.0%)**	**0.002†**			
No	**247/444 (55.6%)**	**12 (100.0%)**				
**Vaccination**						
No	**80 (25.6%)**	**4 (66.7%)**	**0.043‡**	**0.011**	**0.001-0.280**	**0.006**
Yes	**233 (74.4%)**	**2 (33.3%)**				
Vaccination status						
Not vaccinated	76 (23.6%)	4 (66.7%)	0.112‡			
Vaccinated (1 dose)	22 (6.8%)	0 (0.0%)				
Vaccinated (2 doses)	107 (33.2%)	2 (33.7%)				
Vaccinated (3 doses)	112 (34.8%)	0 (0.0%)				
Vaccinated (4 doses)	5 (1.6%)	0 (0.0%)				
Gender						
Female	315 (70.6%)	6 (50.0%)	0.124†			
Male	131 (29.4%)	6 (50.0%)				
Age						
Mean ± SE	**50.9 ± 0.6**	**67.5 ± 5.0**	**0.001⁂**	**1.087**	**1.008-1.172**	**0.031**
SAIRD						
RA	97 (21.7%)	1 (8.3%)	0.475†			
SpA	142 (31.8%)	1 (8.3%)	0.083†			
Other arthritis	14 (3.1%)	0 (0.0%)	0.533†			
SLE / APS	77 / 6 (18.6%)	1 (8.3%)	0.745‡			
SSc	42 (9.4%)	3 (25.0%)	0.074†			
Other CTD	**29 (6.5%)**	**3 (25.0%)**	**0.013†**	9.126	0.715-116.5	0.089
Vasculitis	39 (8.7%)	3 (25.0%)	0.054†			
Other ID	9 (2.0%)	0 (0.0%)	0.619†			
Overlapping	16 (3.6%)	0 (0.0%)	0.504†			
Treatments						
**Glucocorticoids**	**231/424 (54.5%)**	**3/12 (25.0%)**	**0.043†**	0.278	0.030-2.584	0.260
**No treatment**	**26/445 (5.8%)**	**3/12 (25.0%)**	**0.007†**	0.107	0.003-4.348	0.237
**DMARDs**	253/446 (56.7%)	3/12 (25.0%)	0.029†	0.271	0.028-2.625	0.260
Immunosuppressants*	57/446 (12.8%)	3/12 (25.0%)	0.216†			
TNFi	130/446 (29.1%)	1/12 (8.3%)	0.115†			
IL-6i	17/446 (3.8%)	1/12 (8.3%)	0.426†			

IL-17/23/12i	22/446 (4.9%)	0/12 (0.0%)	0.430†			
B cell depletion	34/446 (7.6%)	3/12 (25.0%)	0.029†	3.344	0.291-38.36	0.333
JAKi	13/446 (2.9%)	0/12 (0.0%)	0.549†			
Belimumab	14/446 (3.1%)	0/12 (0.0%)	0.533†			
Other	20/446 (4.5%)	0/12 (0.0%)	0.453†			
Comorbidities						
Diabetes mellitus	36/444 (8.1%)	2/12 (16.7%)	0.290†			
Arterial hypertension	120/444 (27.0%)	5/12 (41.7%)	0.262†			
Malignancy	9/444 (2.0%)	0/12 (0.0%)	0.618†			
**Pulmonary diseases**	74/446 (16.6%)	8/12 (66.7%)	<0.0001†	**9.003**	**1.116-72.62**	**0.039**
Cardiovascular diseases	45/446 (10.1%)	3/12 (25.0%)	0.096†			
Obesity	61/446 (13.7%)	3/12 (25.0%)	0.264†			
Smoking ever	234/397 (58.9%)	8/12 (66.7%)	0.592†			
Other/Unknown	13/444 (2.9%)	0/12 (0.0%)	0.518†			
**None**	160/444 (36.0%)	0/12 (0.0%)	0.010†			

† Chi-square test
‡ Fisher's Exact test
⁑ Log-Rank test
⁂ Student's t-test
⁋ Binary regression
*Includes cyclophosphamide, mycophenolate, cyclosporine
DMARDs include: hydroxychloroquine, methotrexate, leflunomide, azathioprine, sulfasalazine, apremilast
p-values <0.05 are shown in bold
